# *In vivo* evolution of antimicrobial resistance in a biofilm model of *Pseudomonas aeruginosa* lung infection

**DOI:** 10.1093/ismejo/wrae036

**Published:** 2024-03-13

**Authors:** Doaa Higazy, Anh Duc Pham, Coen van Hasselt, Niels Høiby, Lars Jelsbak, Claus Moser, Oana Ciofu

**Affiliations:** Costerton Biofilm Center, Department of Immunology and Microbiology, University of Copenhagen, 2200 N Copenhagen, Denmark; Department of Microbiology, Faculty of Agriculture, Cairo University, 12613 Giza, Egypt; Department of Clinical Microbiology, Rigshospitalet, University of Copenhagen, 2100 Ø Copenhagen, Denmark; Division of Systems Pharmacology & Pharmacy, Leiden Academic Centre for Drug Research, Leiden University, 2300 RA Leiden, The Netherlands; Division of Systems Pharmacology & Pharmacy, Leiden Academic Centre for Drug Research, Leiden University, 2300 RA Leiden, The Netherlands; Costerton Biofilm Center, Department of Immunology and Microbiology, University of Copenhagen, 2200 N Copenhagen, Denmark; Department of Clinical Microbiology, Rigshospitalet, University of Copenhagen, 2100 Ø Copenhagen, Denmark; Department of Biotechnology and Biomedicine, Technical University of Denmark, 2800 Kgs. Lyngby, Denmark; Costerton Biofilm Center, Department of Immunology and Microbiology, University of Copenhagen, 2200 N Copenhagen, Denmark; Department of Clinical Microbiology, Rigshospitalet, University of Copenhagen, 2100 Ø Copenhagen, Denmark; Costerton Biofilm Center, Department of Immunology and Microbiology, University of Copenhagen, 2200 N Copenhagen, Denmark

**Keywords:** Pseudomonas aeruginosa, biofilm, in vivo evolution, antimicrobial resistance, inflammatory response

## Abstract

The evolution of antimicrobial resistance (AMR) in biofilms has been repeatedly studied by experimental evolution *in vitro*, but rarely *in vivo*. The complex microenvironment at the infection site imposes selective pressures on the bacterial biofilms, potentially influencing the development of AMR. We report here the development of AMR in an *in vivo* mouse model of *Pseudomonas aeruginosa* biofilm lung infection. The *P. aeruginosa* embedded in seaweed alginate beads underwent four successive lung infection passages with or without ciprofloxacin (CIP) exposure. The development of CIP resistance was assessed at each passage by population analysis of the bacterial populations recovered from the lungs of CIP-treated and control mice, with subsequent whole-genome sequencing of selected isolates. As inflammation plays a crucial role in shaping the microenvironment at the infection site, its impact was explored through the measurement of cytokine levels in the lung homogenate. A rapid development of AMR was observed starting from the second passage in the CIP-treated mice. Genetic analysis revealed mutations in *nfxB*, efflux pumps (*mexZ*), and two-component systems (*parS*) contribution to CIP resistance. The control group isolates exhibited mutations in the *dipA* gene, likely associated with biofilm dispersion. In the initial two passages, the CIP-treated group exhibited an elevated inflammatory response compared to the control group. This increase may potentially contribute to the release of mutagenic reactive oxygen species and the development of AMR. In conclusion, this study illustrates the complex relationship between infection, antibiotic treatment, and immune response.

## Introduction

Antimicrobial resistance (AMR) is becoming a growing threat to public and environmental health and has been identified as a major hazard in our society. *Pseudomonas aeruginosa* is an opportunistic Gram-negative pathogen with intrinsic resistance to several antibiotics as well as an increased tendency for developing further resistance, and it is one of the major causes of morbidity and mortality in cystic fibrosis (CF) patients [1]. The *P. aeruginosa* is subject to evolutionary adaptation, including the transition into biofilms that induce chronic infection in CF patients paralleled with the production of virulence factors [2, 3], and the evolution of high antibiotic tolerance [4]. The large genome of *P. aeruginosa* (>6 Mb) encodes many regulatory genes responsible for metabolism and resistance mechanisms and controlling the virulence factors, which facilitates bacterial adaptation to various environmental changes and enhances its long-term survival and persistence [5]. The host response to chronic *P. aeruginosa* biofilm infections shows remarkable complexity and variability. It encompasses a spectrum that includes robust but ineffective activation of innate and adaptive immunity against the infecting bacteria and a state of host tolerance, resulting in collateral tissue damage instead of bacterial elimination [6].

In contrast to the planktonic, fast-dividing cells that are traditionally used to study antibiotic resistance development in shaking batch cultures, biofilm-grown bacteria are fundamentally different as they encounter gradients of nutrients and oxygen in a sessile condition, which lead to a heterogenous bacterial population, including slow-growing or nondividing cells [7, 8]. Both planktonic and biofilm bacterial populations can develop resistance through spontaneous or antibiotic-induced mutagenesis, followed by selective pressure and competition between individual mutants with different fitness costs. In planktonic populations, the competition allows the selection of high-level resistant mutants with the lowest fitness cost. By contrast, biofilms develop resistance more diversely than planktonic populations due to their spatial organization, as they can keep low-level and high-level resistant mutants with high and low fitness costs [9, 10].

Several models were developed to simulate chronic infections caused by *P. aeruginosa*, such as the lung infection model that was achieved by infecting BALB/c mice with the *P. aeruginosa* encapsulated in seaweed alginate beads. The *P. aeruginosa* in polymeric hydrogels was found to form typical biofilms and tolerate antibiotic treatment *in vivo* and *in vitro* [11]. By contrast, the synthetic CF sputum medium has been developed as an *in vitro* model to study the infection physiology of *P. aeruginosa* [12] and has been utilized to answer various questions in studying biofilm antimicrobial susceptibility for different antibiotics [13]. In addition, other chronic infection models, such as the chronic wound model, have also been developed [14, 15]. Research has also explored *ex vivo* models, revealing in a pig lung model that pathogens can form biofilms within a structure more clinically realistic than conventional *in vitro* models [16].

The *P. aeruginosa* in patients with chronic infections is characterized by the overproduction of the mucoid exopolysaccharide, alginate [17]. The *P. aeruginosa* can be encapsulated in alginate beads and form clusters resembling the *in vivo* aggregates of chronic infections [18, 19]. In one previous study, *P. aeruginosa* embedded in the seaweed alginate beads were fixed subcutaneously (SC) in BALB/c mice, and all the mice were exposed to tobramycin treatment. This *in vivo* model confirmed that biofilms behave as independent microcompartments also *in vivo*, impacting the pharmacodynamics of antibiotics [20]. Tolerance to antibiotics, distinct from resistance, enables bacterial survival during exposure without conventional resistance mechanisms. This survival can result from genetic factors, such as mutations leading to increased lag time, environmental factors like the production of protective biofilm matrices, or slow growth due to microenvironmental conditions [21].

Previous evolutionary experiments of AMR in *in vitro* biofilms in our laboratory showed a different dynamic of resistance development in biofilms compared to planktonic populations. After seven passages of evolution using the sub-MIC concentration 0.1 mg/l ciprofloxacin (CIP) treatment, a higher ratio of CIP resistance was observed in biofilms compared to planktonic cultures. The biofilm-evolved CIP resistance isolates had lower MICs compared to the planktonic CIP-resistant populations [22]. The role of the reactive oxygen species in mutagenesis caused by the evolved biofilm population under CIP treatment was shown using the *P. aeruginosa* strain lacking the major catalase “Δ*katA*.” A faster AMR development was observed in Δ*katA* populations compared to the wild-type PAO1, which points to the potential role played by oxidative stress in promoting AMR [23]. *In vivo*, the source of oxidative stress is the host immune response represented by polymorphonuclear neutrophils (PMNs), besides antibiotics at subinhibitory concentrations, resulting in intrabacterial accumulated toxic hydroxyl radicals [22].

The complex microenvironment at the site of biofilm infection consisting of the inflammatory response, the local oxygen levels, and the nutrient availability combined with the *in vivo* pharmacokinetic (PK) of antibiotics creates specific selective pressures that might influence the evolution of biofilms [24]. The implications of this complex environment for the *in vivo* evolutionary trajectories of AMR are unclear. Therefore, the objective of the present study was to establish an *in vivo* experimental evolution model to better understand the evolutionary process and AMR development in biofilm infections. The mouse model of chronic *P. aeruginosa* lung infection was used to conduct the experimental evolution study [15]. In this model, *P. aeruginosa* is embedded in seaweed alginate, which resembles the naturally dominant exopolysaccharide of *P. aeruginosa* isolated from chronic infections [25]. The seaweed alginate bead model recapitulates the physical aspects of microbial biofilms in terms of antibiotic tolerance, heterogeneous growth, and hypoxia [19]. We demonstrate that resistance developed rapidly in the CIP-treated mice, which also showed a high inflammatory pattern in the first two passages after treatment compared to control mice treated with injection saline (Placebo) at the same time points. The CIP resistance mutations that occurred *in vivo* during evolution in biofilms were identified by whole-genome sequencing (WGS) of selected isolates.

## Materials and methods

### Bacterial strain and alginate beads preparation

In this study, we used the reporter strain PAO1-*mCherry*-P*_CD_*-*gfp* + (PAO1 background) tagged with *mCherry* for a constitutive red fluorescence and designed to have a chromosomal transcriptional fusion between the P*_mexCD_* Promoter and *gfp*, leading to green fluorescence upon mutation of *nfxB,* which was previously developed in our lab [26]. The MIC for CIP of the original strain was determined by E-test (bioMérieux) (MIC = 0.094 mg/l). Protanal LF 10/60 (IMCD, Helsingør, Denmark) was dissolved in 0.9% NaCl to an alginate concentration of 1% and was sterile-filtered. A single colony was selected from the overnight culture prepared from the frozen stock and was grown overnight to reach an optical density of 2 at OD_600._ The bacterial culture was centrifuged followed by resuspension in a new LB medium (half the volume of the original culture) and mixed with the alginate 1% in a ratio of 1:20. The seaweed alginate beads were prepared using the Encapsulation Unit Nisco Var J30 as previously described [27], and we used the 0.250 mm nozzle with an alginate flow rate of 20 ml/h, and the pressure was set at 35 mBar. Afterward, the beads were stabilized for 1 h in the gelling bath of 0.1 M Tris–HCL and 12.5 mM CaCl_2_, then washed twice with (0.9% NaCl containing 0.1 M CaCl_2_). The beads were plated to determine the bacterial counts CFU/ml and the stock was stored overnight at 4°C (Day 0). The minimal biofilm inhibitory concentration (MBIC) for the strain was determined *in vitro* by exposing the bacteria embedded in alginate beads to different concentrations of CIP each in a separate tube and was incubated overnight in a shaker at 37°C for 24 h. The beads were then washed and dissolved using citrate buffer, serial dilutions were made from each concentration, and they were plated on LB agar. The MBIC was determined at 0.5 mg/l CIP concentration, which was found to decrease the CFU by six logs compared to the nontreated biofilms (from 10^9^ CFU/ml to 10^3^ CFU/ml).

### Animals

Female 11-week-old BALB/c mice (*n* = 72 evolution study), (*n* = 40 PK study), and (*n* = 5 Background mice) were purchased from Janvier Labs (Rte. du Genest, 53,940 Le Genest-Saint-Isle, France). The mice were allowed to acclimatize for 7 days before use in the rodent facility at the Biotech Research & Innovation Centre, University of Copenhagen. All the mice had free access to chow and water and were observed daily. The animals were kept in an environment characterized by a 12-h light–dark cycle and temperature and humidity control. The mice were assessed daily to monitor the development of the disease. To ensure a consistent evaluation, each animal was scored individually, and the scoring was done by the same researcher. In each passage, we selected a sample size of 10 mice for the CIP-treated group and 8 mice for the saline-injected control group. Out of the 72 mice, 54 mice were included in the statistical analysis, and the differences in mice numbers in the data included are either due to bacterial clearance in the lungs (no CFU counts) or the mortality of the mice that occurred on the day of the tracheotomy (either during surgical procedures or in the early stages of postoperative recovery, as they failed to regain consciousness); license nr. 2019-15-0201-00183.

### Pharmacokinetics of ciprofloxacin exposure in the lung

BALB/c mice *n* = 40 were injected with 1 mg (45.5 mg/kg) x1 CIP SC (CIP, Fresenius Kabi 2 mg/ml infusion solution, Uppsala, Sweden). The mice were euthanized at different time points (0, 15 min, 30 min, 1 h, 2 h, 4 h, 8 h, and 24 h), and lungs were collected in 1 ml of ice-cold PBS. The lungs were homogenized using MagNa Lyser (Roche), and they were diluted 1:3 with acetonitrile and well vortexed. Then, the lungs were centrifuged, and the supernatant was carefully transferred to a new tube. The determination of CIP concentrations was done following a biological method developed by our lab [28]. The concentration was determined concerning the CIP-sensitive strain *Proteus rettgeri* D9228/71. An overnight culture of the sensitive strain was diluted to 10^−4^ in LB, 2 ml was poured on a blood agar plate to cover the whole plate, the extra liquid was removed, and the plates were allowed to dry at 37°C for 30 min. Standard concentrations of CIP ranging from 0.1 to 33 mg/l were prepared and a number of holes were made in the dried plates, which were filled with different standards and lung samples. The plates were set for 1 h and were then incubated overnight at 37°C. The inhibition zones were measured and used to make both standard and PK curves for different time points of CIP in the lung.

### Lung infection and treatment

By the day of lung infection, the alginate beads bacterial mixture is adjusted to 10^7^ CFU/ml beads in all different passages. The overnight culture was mixed with alginate in a ratio of (1:19) and passed through a nozzle to form small beads size of 40 μm [27]. The CFU inside the beads was determined, and a total CFU of 10^7^ was used in all different passages. On Day 1, the mice have anesthetized SC with a mixture of 1:1:2 (25%) a cocktail of hypnorm (fentanylcitrat, 78.75 mg/l; fluanisone, 2.5 mg/ml) combined with (25%) midazolam (1.25 mg/ml) and (50%) injection sterile water (10 ml/kg body weight). Under anesthesia, the BALB/c mice were tracheotomized, and a curved-tipped needle of 40 μl alginate beads was directed into the left lung, and the incision was then sutured to allow healing [29]. The mice were maintained in a warm environment and were closely observed until they regained consciousness. Subsequently, they received a subcutaneous injection of 1 ml saline and were administered analgesic buprenorphine (Temgesic 0.3 mg/ml) for pain management.

On Day 2, the mice were scored and randomly divided into two groups, a group that was treated with CIP 0.25 mg (11.35 mg/kg) ×2 CIP with an 8-h interval, and a control group that received saline at the same time points. On Day 3, the mice were euthanized, and the lungs were collected. Bacteria from the homogenized lungs were used for population analysis. The surviving resistant colonies, capable of growth on CIP-supplemented plates, were selected and used to initiate an overnight culture to make alginate beads for infecting the next passage. The background mice were used in this study to determine the inflammatory effect that might have been induced by the presence of the beads in the lungs, and the mice were tracheotomized and were injected with alginate beads but free of bacteria.

### Bacteriology and population analysis

The whole lung of the mice was obtained aseptically on Day 3. The lungs were added to a 1 ml 0.9% saline and homogenized using MagNaLyser, two times at 6000 for 10 s. Seaweed alginate bacterial beads were dissolved in citric buffer with a 50× dilution, and then serial dilution was prepared and plated with a triple determination for bacterial counts. We prepared plates of Pseudomonas isolation agar (Fisher Scientific, UK) supplemented with different CIP concentrations (0, 0.5, 1, and 2 mg/l) for population analysis. The number of resistant colonies on each CIP-containing plate was compared to the whole population growth CFU on CIP-free plates to calculate the percentage of the resistant population.

### Evolution experiment

The bacteria obtained from the lungs were plated on Blue agar [30] for CFUs and on Pseudomonas isolation agar supplemented with CIP (0, 0.5, 1, 2 mg/l) for population analysis. We optimized selection criteria for the colonies that will be chosen after the first passage to start an overnight culture for making beads for the new passage. The selection was based on the colonies that were capable of growth on the highest concentration of CIP-supplemented plates. For the CIP-treated group, the colonies were selected from 0.5 mg/l plates (no growth shown on 1 mg/l) after the first passage to start an overnight culture for the second passage. From the second to the third passage, the colonies were selected from 1 mg/l CIP plates. From the third to fourth, they were selected from 2 mg/l CIP plates. This strategy was followed for both treated and control groups; however, there was no growth of any colonies on CIP plates in all passages for the control group, which is why the colonies were mixed from the CIP-free plates to start a new passage for all control passages (Fig. S1).

### Microscopy

The blue plates [30] (CIP-free) are used for quantitative bacteriology in the lung. Images were captured for different CFU plates from different individual mice in different passages of both treated and control groups. The colonies green/red (*gfp*/*mCherry*) were visualized using a Zeiss AxioZoom V16 Microscope (Carl Zeiss) after adjusting the number of tiles to cover the whole plate area. At least three images were captured for each plate, the detectors were optimized for detecting GFP fluorescence (green) (excitation at 488 nm and emission peak at 517 nm), and mCherry (red) (excitation at 594 nm and emission peak at 610 nm). The percentage of *nfxB* mutants was calculated by dividing the number of green colonies by the total CFU counts for each individual mouse lung.

### Cytokine measurements

The measurement of cytokines in the supernatant of the lung homogenates was done by Luminex multiplex analysis (Mouse Magnetic Luminex Assays, R&D systems, Abingdon, UK). The measurements were performed in duplicates with the supernatant from the lung homogenates of the infected and control mice groups according to the manufacturer’s instructions. The samples were diluted 2-fold for (IFN-γ, IL-5, TNF-α, IL-1β) and 20-fold for (G-CSF, CXCL2) measurement, and the plates were read in a Luminex 200 Platform (Luminex Corp., Austin, TX, USA).

### Whole-genome sequencing

A total of 69 isolates originating from the two studied groups of mice: the CIP-treated group (T) (48 isolates), and control mice (C) (20 isolates), were collected as illustrated in the population analysis (Fig. S1). In short, the colonies were selected from CIP plates with different concentrations. Different colony morphologies with different MICs from each mouse were represented in each passage. DNA was isolated using DNeasy Blood & Tissue kit (Qiagen, Netherlands) according to the manufacturer’s instructions. Libraries were produced following Hackflex workflow [31], and libraries were sequenced on a NovaSeq 6000 instrument. The sequenced reads were checked for quality by FastQC and were aligned to the reference PAO1 (GenBank accession no. NC_002516) using bwa-mem and SAMtools [32]. Freebayes was used to call the single-nucleotide polymorphisms, multinucleotide polymorphisms, and insertion and deletions. All the variants were filtered with QUAl > 20 and DP >10 using BCFtools and were finally annotated with Snpeff [33]. Descriptive information for different isolates observed mutations is available in Table S3. The output data from Snpeff can be found in the supplementary datasets [1-8].

### Growth curves and minimal inhibitory concentrations determination for sequenced isolated

Overnight cultures of bacterial isolates in LB were adjusted to 0.1 OD_600_ and diluted 1000-fold, and 100 μl of each isolate dilution were transferred in triplicates in a 96-well plate with a flat bottom (Nunc, Thermo Fisher Scientific), which were incubated in Infinite F200 Pro plate reader (Tecan) with lid on, at 37°C and shaken at 225 rpm for 24 h. Absorbance (OD_600_ nm) was measured using Magellan V 7.2 software, every 20 min during 24 h incubation. Growth curves were constructed and were used to calculate the growth rate and lag phase using the Gompertz model. For MICs determination, the overnight cultures were diluted 10 000 fold and 1 ml of the diluted culture was poured onto a blood agar plate, the excess liquid was discarded, and the plates were allowed to dry and E-test strips (bioMérieux SA, France) were fixed in the middle of each plate and were incubated overnight in 37°C.

### Statistics

Unpaired *t*-tests were used to analyze data (Prism 9, GraphPad Software, San Diego, USA). R (v4.2.1) was used for data processing, statistical tests, and visualization. PK analysis of CIP was performed using nlmixr (v2.0.7) [34]. A *P*-value ≤ .05 was considered to be statistically significant. The growth rate for the different isolates was calculated using the below equation. To compute growth rates and lag times, we fitted a Gompertz model to the individual growth curves for each biological replicate individually using R (version 4.2.1). The following equation was used [35].


$$ {\mathrm{OD}}_t={\mathrm{OD}}_{\mathrm{t}0}+{\mathrm{OD}}_{\mathrm{max}}\times{e}^{-{e}^{\left(\frac{e\cdot{\mathrm{k}}_{\mathrm{gr}}}{\ {\mathrm{OD}}_{\mathrm{max}}}\left({t}_{\mathrm{lag}}-t\ \right)+1\right)}}. $$

Here, *t*_lag_ denotes the lag time, k_gr_ represents the absolute growth rate, OD_*t*0_ denotes the optical density measured at the start of the experiment, and OD_max_ indicates the optical density at the asymptote. Adequate fits were obtained (Fig. S2), and the mean values of fitted growth parameters across replicates were subsequently calculated.

### Pharmacokinetics modeling and simulation

PK modeling was conducted in R (v 4.2.1) using the nonlinear mix effect modeling R package nlmixr (v2.0.7) [34]. Parameter estimation was performed using the first-order estimation method. An empirical one-compartment model with absorption, clearance, and distribution was used to describe the data. The developed PK model was used to simulate the dosing regimens for the *in vivo* experiments using the R package RxODE (v2.0.7) [36].

## Results

### Animal model used for experimental evolution of antimicrobial resistance

**Figure 1:**
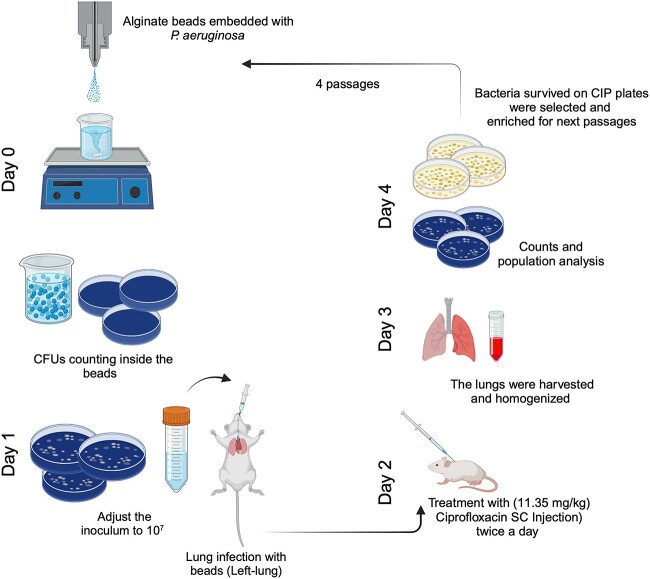
The experimental setup of the evolution of *P. Aeruginosa* in a biofilm lung infection model in BALB/c mice; the experimental setup started with an overnight culture from a single colony of PAO1-*mCherry*-P*_CD_*-*gfp*+; on Day 0, bacteria were embedded in alginate beads; on Day 1, mice were infected by injecting the left lung with alginate beads; on Day 2, the mice were treated twice with either CIP 0.25 mg (11.35 mg/kg) ×2 or saline (Placebo); on Day 3, the mice were euthanized, and the lungs were collected, homogenized, and used for population analysis; on Day 4, colonies from CIP plates were selected for overnight cultures that were embedded in new alginate beads to infect a new group of mice (a new passage); the illustration was created with BioRender.com

The experimental setup of the *in vivo* evolution (Fig. 1) was based on a mouse model of chronic *P. aeruginosa* lung infection [15]. We conducted four passages of biofilm infection utilizing a *PAO1-mCherry-PCD-gfp +* reporter strain [26]. This strain serves as a fluorescent transcriptional reporter specifically designed for the detection of *nfxB* mutants, where *nfxB* functions as the negative regulator of the MexCD-OprJ efflux pump. Mice were lung-infected with bacteria contained within alginate beads, and 24 h following infection, they received two treatments of either 0.25 mg (11.35 mg/kg) ×2 of CIP or saline over the course of 1 day; 48 h following infection, all the mice were euthanized, bacteria were collected from the lungs, and population analysis was conducted. Bacterial colonies exhibiting growth on the highest concentration of CIP (0.5, 1, and 2 mg/l) were selected, embedded in alginate beads, and used to infect the lungs of a new group of mice.

### Pharmacokinetics of ciprofloxacin in lungs

**Figure 2:**
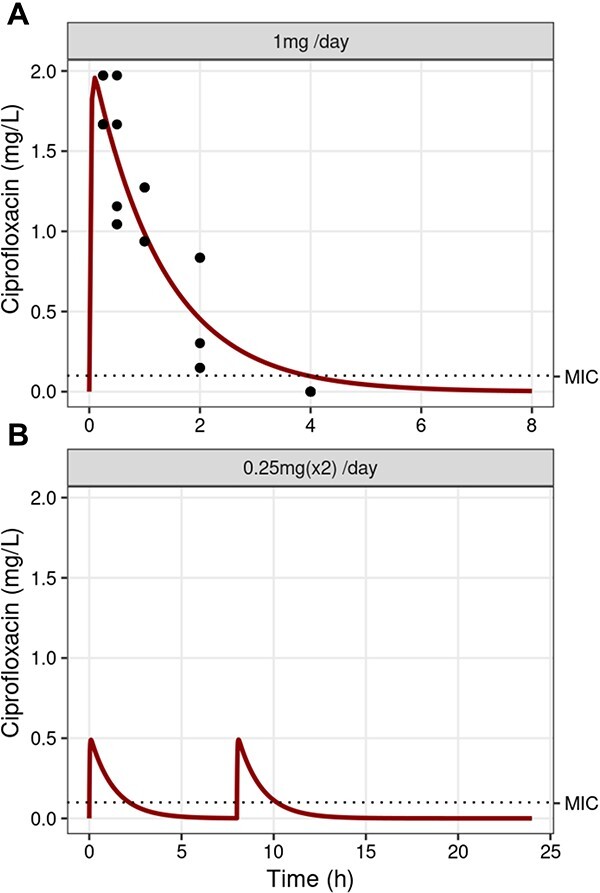
Drug PK and modeling; PK of CIP in the mouse lung with the MIC indicated; (A) PK model fit of CIP dosed 1 mg (45.5 mg/kg) ×1 (SC using a one-compartment model, model prediction (red lines), and the observed data (points) following SC administration of 1 mg CIP); (B) simulations for concentration–time courses in the lung for treatment dosing regimens of 0.25 mg (11.35 mg/kg) SC CIP at 8-h intervals; the corresponding dosing schedule is presented in the header of the figure; the dotted line represents the MIC of 0.094 mg/l.

A single-dose lung PK study was performed, given a SC dose of 1 mg (45.4 mg/kg) ×1 CIP. The observed average maximum concentration of CIP C_max_ was 1.78 mg/l of homogenized lung tissue with an area under the curve = 2.398 as a measure of drug exposure (Fig. 2a). PK modeling was conducted using a one-compartment model, and the estimated parameters presented in Table S1. The model adequately describes the concentration-time profile (Fig. 2). Pilot animal studies were performed to establish a CIP dose that maintains the *P. aeruginosa* alginate beads infection in the lungs of the mice (Fig. S3), and we found that 0.25 mg/mouse administered twice was suitable for the evolution studies (the bacteria were not eliminated from the lung). Using the PK model (Fig. 2a), we performed simulations of the *in vivo* dose regimen (Fig. 2b), 0.25 mg (11.35 mg/kg) ×2 which led to CIP peak concentrations of 0.5 mg/l, i.e. at 5× the strain MIC and equal to MBIC. This concentration is in the range of CIP concentration achieved in the sputum of patients with CF 4–8 h after an oral administration of 500 mg CIP [37].

### Lung bacteriology and ciprofloxacin resistance development during experimental evolution

**Figure 3:**
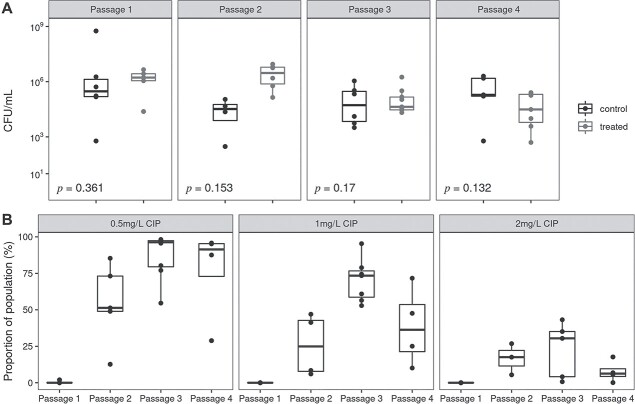
Bacterial load and population analysis in lung homogenates; (A) the bacterial load in the lungs (CFU/ml) of CIP-treated and control mice from the different passages (Passages 1–4); no statistically significant differences were observed between the two groups of animals (*P* > .05, *t*-test) in either of the passages; (B) results of the population analysis of the bacteria in the lungs of treated animals in the different passages [1-4] presented as a fraction of the bacterial population that survived on plates with different CIP concentrations (0.5, 1, and 2 mg/l); significant differences were observed between the first passage compared to other passages (*t*-test, *P* < .05).

To investigate the development of CIP resistance during this experimental evolution study, we conducted population analysis of the bacterial populations from the mouse lungs from the different passages by plating the lungs homogenate on LB agar plates containing CIP in different concentrations (0.5, 1, and 2 mg/l). The bacterial load in the lung homogenates of treated and control groups was measured, and we found similar bacterial counts (CFU/ml) between the two groups in the different passages (Fig. 3a). The bacteria obtained from the control mice lungs did not grow on CIP plates during different passages as illustrated in Fig. S1. The fraction of the bacterial population surviving different CIP concentrations in different passages revealed an increase in early passages (Fig. 3b). The bacteria from the treated lungs gained resistance early starting from the first passage that significantly increased when compared to the second, third, and fourth passages (*P* = .045, 0.0001, and 0.0142, respectively Fig.S4a–c and Table S2). However, no significant differences were detected between second, third, and fourth passages.

**Figure 4:**
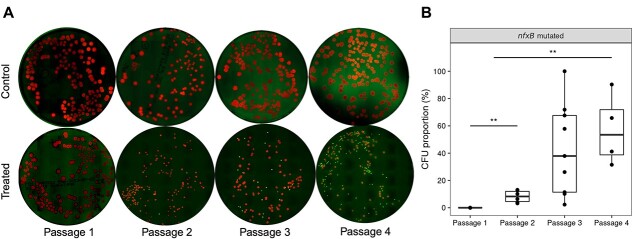
*nfxB* mutants tracked by fluorescence imaging during the evolution experiment; **(**A) the fluorescence of PAO1-*mCherry*-P*_CD_*-*gfp* + colonies of the bacterial populations from lungs of CIP-treated and control mice plated on LB agar plates without CIP; red fluorescence indicates the wild-type and green fluorescence indicates *nfxB* mutants; the ratio of the green colonies to red colonies in the bacterial population from different individuals was calculated and presented in (B), which shows the percentage of *nfxB* mutants in the entire bacterial population in the different passages; ^**^ first and second passages indicate *P* = .0017, whereas ^**^ first to fourth passages indicate *P* = .0019; fluorescence images were captured by a Zeiss AxioZoom microscope; at least three images were randomly captured per petri dish plate; the objectives and detectors were optimized for detecting GFP fluorescence (green) and mCherry fluorescence (red); the number of tiles was adjusted to cover the whole plate area in one image.

The fluorescent reporter strain was constructed by tagging wild-type strain PAO1 with *mCherry* at the chromosomal *attB* (red fluorescence) and then introducing the mini-Tn*7* construct harboring P*_CD_*-*gfp* + (green fluorescence) [26]. The *nfxB* mutants were tracked by visualizing the green fluorescence and calculating the ratio of the *gfp* green (*nfxB* mutants) colonies to the *mCherry* red (wild-type) colonies in the bacterial population (Fig. 4a and b). Fluorescence images were captured for the colonies on CIP-free plates, and the results demonstrated the absence of *gfp* colonies (*nfxB*) within the bacterial populations obtained from the control mice homogenized lungs (Fig. 4a). Thus, the green colonies indicating *nfxB* mutants were only observed on the bacterial counting plates with homogenized lungs from the CIP-treated group. The ratio of the *nfxB* to the whole bacterial population was counted for the different individual mice lungs from the CIP-treated group in different evolutionary passages (Fig. 4b). The results show a significant increase in the ratio of the *nfxB* mutants that was elevated to a mean percentage of 8% in the second passage (*P* = .0017), and 60.6% fourth passage (*P* = .0019) (Fig. 4b).

### Genetic basis of ciprofloxacin resistance during the *in vivo* experimental evolution and phenotypic characterization

**Figure 5:**
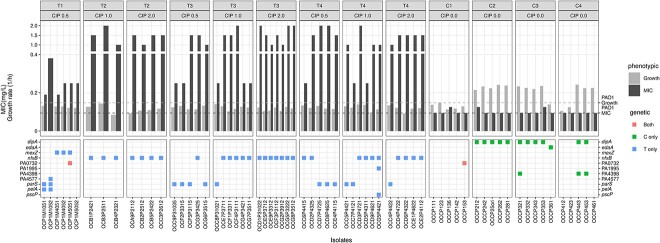
Genetic-phenotype overview; the selected isolates from the treated (T) and control (C) groups of mice in each passage (first row); from different concentrations of CIP plates ranging from 0.5,1.0, and 2.0 mg/l (second row), three to six bacterial isolates were purified per passage (as indicated on the *x*-axis label); for these isolates, the top panel shows both the MIC values (black bars) and the growth rate (gray bars); values for the parental PAO1-*mCherry*-P*_CD_*-*gfp* + are shown in the horizontal dashed lines; the bottom panel shows mutations identified in the genome sequence of the different bacterial isolates in green color for C, blue for T, and red for shared mutations; detailed information on variant annotations is shown in Table S3.

To identify the genetic basis of CIP resistance that occurred during the *in vivo* experimental evolution, we selected bacterial isolates originating from treated (T) and control (C) mice from each passage and we analyzed their WGS. To investigate the fitness cost of the selected isolates, MIC measurements and growth curves in LB were conducted. A full overview of the selected isolates, the concentration of the CIP plate from which the isolates were collected, the MICs toward CIP, their growth rate, as well as all the different genes found to be mutated in the selected isolates are represented in (Fig. 5 and Fig. S2, and Table S3). The chosen isolates epitomize the diverse spectrum of bacteria sourced from individual mice across successive passages, showcasing distinct MICs and colony morphologies. In the T group, mutations were found as a frameshift deletion in *nfxB*, the negative regulator of the MexCD-OprJ efflux pump which was found in 100% of the isolates of Passage 2, 72% of Passage 3, and 62.5% of Passage 4. The sensory histidine kinase *parS* gene exhibited a missense variant in 40% of the first passage isolates, and a deletion mutation in the third and fourth passages of the T group isolates (27.7% and 37.5% respectively) (Fig. 5) and (Table S3). Isolates that had mutations in *nfxB* in the third passage of the T group had a higher MIC ranging between (0.25–2 mg/l) than the *parS* mutants (0.25–1.5 mg/l) (Fig. 5). The analysis of the growth curves showed that the CIP-treated isolates had a slower growth rate than the control isolates starting from the second passage, suggesting a fitness cost of these mutants (*P* < .0001) (Fig. S5a). Isolates harboring the *nfxB* mutation were found to lack the *parS* mutation, and conversely, those with the *parS* mutation did not exhibit the *nfxB* mutation. To explore the potential impact of these mutations on growth, the growth rates of isolates from the third passage treated with CIP were compared. Results revealed that isolates with *parS* mutants exhibited a faster growth rate compared to those with *nfxB* mutants in the third passage (*P* = .0324) (Fig. S5b). Three isolates from the first passage in the T group presented deletion mutations in *mexZ* which is a transcriptional repressor of the MexXY efflux pump. Among the three isolates, one isolate displayed a frameshift deletion, whereas the other two demonstrated a bidirectional gene fusion deletion. Mutations in *pelA* were identified in two independent isolates from the first passage of the T group. *PelA* is an enzyme involved in the biosynthesis of polysaccharides and plays a key role in biofilm formation. A solitary isolate in the second passage revealed a missense variant in the (PA4577) gene, known for its involvement in the RNA polymerase binding transcription factor *dksA*. This factor plays a pivotal role in bacterial adaptation to demanding environmental conditions [38]. A conservative inframe insertion was found in the *pscP* gene that significantly contributes to the Type III secretion system. The isolates selected from the T group had 74% genetic variants in common between different passages. In comparison, the isolates selected from the C group had 64.3% genetic variants in common between different passages (Fig. S6).

In the C group, the selected isolates mainly presented a frameshift variant in the *dipA* gene which encodes for a sensory box/GGDEF family protein, which was found in 100% of the isolates in the second passage and in 80% and 40% in the third and fourth passages, respectively (Fig.5 and Table S3). The GGDEF plays a key role in the cyclic-di-GMP signaling that regulates bacterial biofilm formation, motility, and virulence [39]. In addition, a mutation in (PA4398), which modulates swarming motility and biofilm formation in *P. aeruginosa*, was identified in a single isolate of the third passage and two isolates of the fourth passage in the C group.

In a single isolate from both T and C groups, we identified a conservative inframe insertion in the gene ID (PAO732) which plays a role in L, D transpeptidase (LDTs), which is essential for the peptidoglycan polymerization in biofilms. It has been shown that the deletion of LDTs could impair biofilm formation and stability [40]. The MICs of the selected isolates from the T group are significantly higher than that of the C group (*P* < .0001) (Fig. S7).

### Inflammatory response during *in vivo* experimental evolution

As the inflammatory response is an important player in the shaping of the microenvironment at the infection site, we decided to characterize macroscopically the lungs of the infected animals and to measure the cytokine levels in the lung homogenates at the different passages.

**Figure 6:**
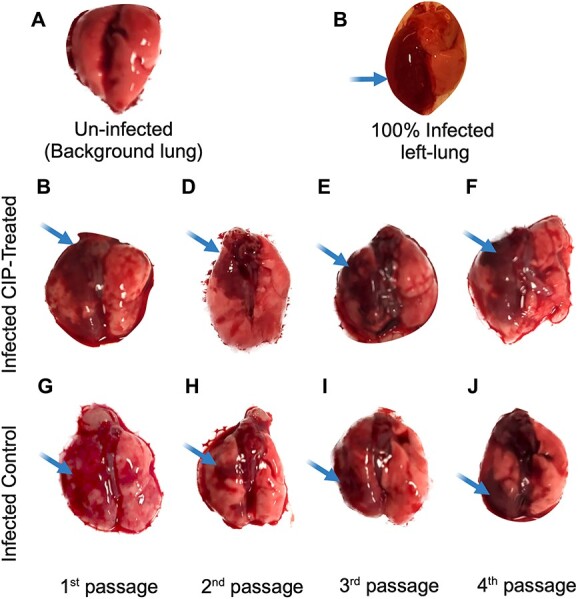
Macroscopic examination of infected and uninfected lungs**;** the figure shows differences in macroscopic pathology between (A) uninfected lungs that were inoculated by bacteria-free alginate beads (background) and infected lungs that were inoculated by *P. Aeruginosa* embedded in seaweed alginate beads (10^7^CFU/ml); inflammation (marked with arrows) was observed in both the CIP-treated mice with CIP (C–F), and the control mice (G–J) in the different passages of the evolution experiment.

Macroscopic examination shows the difference between uninfected lungs (Fig. 6a) and the inflamed left side of infected lungs (Fig. 6b) obtained from a pilot study. The signs of inflammation are observed, as condensed areas representing atelectasis, in the different passages of both treated (Fig. 6c–f) and control mice (Fig. 6g–j). The background mice (uninfected) that received bacteria-free seaweed alginate beads, did not show macroscopic signs of inflammation when compared to the infected ones, which indicates that the inflammation was caused by the bacterial infection and not the beads per se inside the lung.

**Figure 7:**
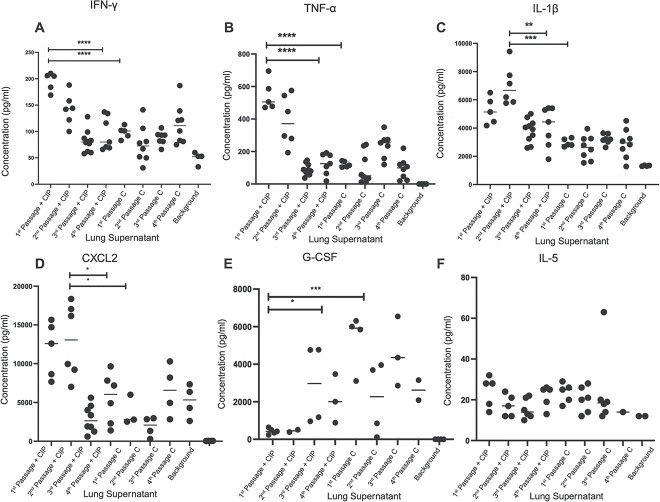
Measurement of cytokines in lungs; the concentration (pg/ml) of cytokines (A) IFN-γ, (B) TNF-α, (C) IL-1β, (D) CXCL2, (E) G-CSF, and (F) IL-5 in the supernatant of the lung homogenates of CIP treated (+CIP) and control mice (C) during the four passages; all the results are compared to the background mice which were inoculated in the lung with alginate beads without bacteria; ^*^P < .05, ^**^P < .01, ^***^P < .001, ^****^P < .0001.

Cytokines IFN-γ, TNF-α, IL-1β, and CXCL2 (Fig.7a–d), and G-SCF and IL-5 (Fig.7e and f) were measured in the supernatant of the lung homogenate of mice at the different passages. The results indicated that four of the cytokines (Fig. 7a–d) followed a similar trend, where the CIP-treated group in the first two passages showed the highest cytokine levels compared to the third, fourth, and all control passages. Specifically, IFN-γ and TNF-α (Fig. 7a and b) were highly expressed in the first passage for CIP treated group compared to the control group (*P* < .0001), followed by a reduction in the concentrations of the cytokines in the third and fourth passages of the CIP-treated groups (*P* < .0001). Similarly, IL-1β and CXCL2 were most elevated in the second passage of the mice lung from the CIP-treated group (Fig. 7c and d). G-CSF level showed an increase in the third and fourth passages compared to the initial two in the CIP group (Fig. 7e). Additionally, the results show a significantly higher level of G-CSF in the first passage of the control mice compared to the CIP-treated one, which indicates a stronger PMN recruitment in the control mice, in contrast to a possible stronger mononuclear cells MNCs cell activation in the CIP-treated group. No pattern was observed for the control group. IL-5 showed no significant differences between the different passages (Fig. 7f).

## Discussion

We present an experimental evolution study of AMR in an *in vivo* mouse *P. aeruginosa* biofilm lung infection model. The model enabled the investigation of bacterial mutations that emerged during evolution under both CIP treatment and control conditions and associated production of proinflammatory cytokines during the infection.

In accordance with the *in vitro* studies [22, 23, 41], a fast development of resistance was observed in this *in vivo* experimental evolution during CIP treatment of mice with biofilm lung infection. The development of AMR was accompanied by mutations in nfxB with the accumulation of gfp-expressing mutants of *PAO1-mCherry-P_CD_-gfp +* during the different passages and this is in accordance with previous *in vitro* studies in flow-cells [26]. These findings are in alignment with results from experimental evolution studies in biofilms conducted with other microorganisms in different biofilm models supporting common evolutionary trajectories in biofilms [21]*.*

The genetic basis of *in vivo*-evolved CIP-resistant isolates identified mutations in genes regulating the efflux pumps (*nfxB* and *mexZ*), in addition to mutations in genes encoding for *parS*, *pelA*, and *pscP*, which have been reported previously in the *in vitro* CIP-evolved isolates [41]. ParS is the sensor component of the two-component ParS/ParS system which has been shown to be involved in the expression of the MexEF-OprN efflux pump that exports quinolones [42]. In addition, this two-component regulatory system was shown to play a role in regulating the quorum-sensing system in *P. aeruginosa* [42], indicating the pleiotropic indirect effects of resistance development.

*In vivo*-specific mutations, such as that was found in (PA4577) encoding the RNA polymerase-transcription factor *dksA*, were observed in the treated group of mice (Fig. 5). DksA is a transcription factor that regulates the stringent response which is recognized to be involved in the regulation of bacterial virulence and metabolism. In isolates from control *in vivo* biofilm infection (controls), we identified mutations in *dipA* (sensory box /GGDEF family protein), sensory box histidine kinase, and *edaA* (carbohydrate-selective porin OprB), which were not reported in the *in vitro* biofilm evolution studies. DipA is a phosphodiesterase that is essential for *P. aeruginosa* biofilm dispersion [43], suggesting the *in vivo* selection of mutants causing persistent biofilms. Similar findings were also observed by a study that tested chemical compounds for their ability to biofilm dispersal, and they indicated that H6–335-P1 was capable of dispersing *P. aeruginosa* formed biofilms by *dipA*, *rbdA*, *mucR*, and *nbdA* mutants [44].

Similar to what has been previously observed in *in vitro* evolution experiments [21-23, 41], we observed that CIP treatment selects not only for adaptive mutations in AMR genes (*nfxB*, *parS*) but also in genes involved in general adaptation, such as biofilm formation (*pel*), virulence (Type III secretion) (*pscP*), or metabolism (*dskA*), supporting the role played by antimicrobial treatment in adaptive evolution in biofilms. In general, the findings from this *in vivo* evolution experiment in a mouse biofilm lung infection model reproduce the results of *in vitro* experiments supporting selection in biofilms exposed to antibiotics of phenotypes with increased expression of efflux pumps and impaired metabolism [21]. No mutations in the quinolone-resistance determinants such as *gyrA*, *gyrB*, or *parC* were found in the isolates from the treated group. This aligns with our previous results, showing that the biofilm mode of growth promotes low-level resistance mutations compared to planktonic growth [22, 41, 45]. This can be explained by the short-term treatment of the biofilm infection in this model (only two administrations at 8-h intervals) in each passage that is probably insufficient to reach the steady-state PK in the mouse lungs [46].

The main components of the innate immune response engaged in response to *P. aeruginosa* biofilm include neutrophils, and an intense accumulation of activated neutrophils in the airways occurs in the lungs of mice infected with alginate beads [9]. In acute infections, clearance of planktonic bacteria occurs due to the combined activation of the innate and adaptive immune systems. However, in the case of chronic biofilm infections, the pathogens are not eliminated and the ineffective host response (innate and adaptive) is considered to be the cause of the biofilm-related pathology [47]. The infected mice in the present experimental evolution exhibited manifestation of lung inflammation, and this was in contrast to the lungs of mice where bacteria-free alginate was instilled, showing that the inflammatory response is directed toward bacterial products and not toward the seaweed alginate.

One mechanism by which *P. aeruginosa* evades the immune system includes the release of extracellular products, such as proteases, toxins, and lipases. The measurement of the concentrations of the proinflammatory cytokines (IFN-γ, TNF-α, IL-1β, and CXCL2), revealed a higher concentration in the initial CIP-treated passages compared to the third and fourth treated passages and to all the control passages (Fig.7a–d). This suggests that the CIP treatment applied in this experimental setup stimulates the inflammatory response in the early passages.

TNF-α and IL-1β are proinflammatory cytokines involved in the induction of inflammatory reactions and both are released primarily by macrophages and monocytes during cell infection and inflammation [48]. However, the IFN-γ, which is a critical component of cell-mediated immune response to intracellular pathogens, is mainly produced by activated natural killer cells NK cells, CD4^+^ th1 cells, and CD8^+^ cytotoxic T cells [49]. CXCL2 is a neutrophil chemoattractant produced by tissue macrophages in response to lipopolysaccharides, which is the major component of outer membrane of Gram-negative bacteria [50]. GCSF is stimulating the PMNs from the bone marrow, which is mainly produced by endothelial cells and fibroblasts in addition to monocytes and macrophages [51].

We suggest that the increased inflammatory response in the first passages is due to the increased expression of proinflammatory molecules as a stress response of the *P. aeruginosa* to the presence of a sub-MIC concentration of CIP [52]. It is known that sub-MIC concentrations of CIP induce Pf4 prophages [53] and that Pf4 prophage can become superinfective and induce cell lysis [54, 55], leading to liberation at the site of infection of virulence factors that might be recognized by the immune cells inducing an inflammatory response. The reduced inflammatory response observed in the last two passages of the CIP-treated mice is at similar levels compared to the placebo-treated controls. This might be explained by the development of CIP-resistant *P. aeruginosa* isolates that do not recruit anymore a stress response at the respective CIP concentration.

We observed a reciprocal pattern between the levels of G-CSF and the other cytokines, including IFN-γ, TNF-α, IL-1β, and CXCL2, suggesting a dynamic interplay between the regulation of granulocytes and MNCs during the inflammatory response. G-CSF, a key regulator of granulocytes, has been shown to exhibit a reduced need in the presence of increased levels of IFN-γ, TNF-α, IL-1β, and CXCL2. This may signify a shifting balance between the immune response, with implications for lung inflammation. A decreased reliance on PMNs and a more mononuclear-dominated inflammatory response early on in the CIP-treated group is an intriguing finding that needs further investigation.

In an earlier study that agrees with our model, the *Acinetobacter baumannii* was passaged 15 times in a mouse model of acute pneumonia [56]. That paper discussed whether neutrophils could suppress resistance to CIP by comparing their findings in immune-competent and immune-depleted mice. The percentage of resistant bacteria in the population obtained from immune-competent mice was in a similar range as a function of CIP concentration (up to 2 mg/l) as our results. However, the survival fraction during the population analysis was much higher in our study, and this could be due to the selection bottleneck we introduced in our study. We experienced faster development of AMR when compared to the chronic wound infection model using *P. aeruginosa* which first showed resistance to CIP after 14 days [46]*.* The experimental evolution mouse model allowed an extended growth of *P. aeruginosa* inside the lung, 48 h after infection and 24 h following CIP treatment, which could enable us to detect changes in the interaction between the bacterial and host responses in consecutive passages. The *P. aeruginosa* lung infection in CF patients is maintained with the help of a surrounding polysaccharide matrix (alginate) produced by the bacteria, which inhibits their clearance from the lungs [57]. Alginate beads-embedded PA were used previously as a reproducible model *in vitro*, where they stay stable for a long period and form *in vivo*-like aggregates [19].

Although we consider our model suitable for the investigation of the *in vivo* evolution of resistance in biofilms, there are some limitations, such as the limited number of passages and the relatively short treatment period (two administrations/24 h). An extended phenotypic characterization of the collected *P. aeruginosa* isolates from this *in vivo* evolution experiment would answer the question of the selected phenotypes during CIP treatment of *P. aeruginosa* biofilm infections and will be the subject of future investigations.

In conclusion, this study allowed investigation of the dynamics of biofilm AMR resistance development experimentally *in vivo* in a biofilm lung infection mice model. The fast development of AMR and increased release of cytokines under CIP treatment highlighted the future important focus that should be directed toward the use of antibiotics with considering their complex relationship with the host immune response in addition to their main role as bactericides.

## Supplementary Material

supplementary_material_wrae036

## Data Availability

The datasets acquired from this study are available within the publication and the attached supplemental files. All the data for the sequencing results can be accessed through the SRA project nr: PRJNA1040750.
