# Supplementary material for: In vivo evolution of antimicrobial resistance in a biofilm model of Pseudomonas aeruginosa lung infection
Source: ISME J. 2024 Mar 13;18(1):wrae036. doi: 10.1093/ismejo/wrae036 (PMC10980832; doi:10.1093/ismejo/wrae036)
Supplement: supplementary_material_wrae036 [file supplementary_material_wrae036.zip › Supplementary figures and tables.pdf]

Description	Parameter	unit	Estimate	(RSE%)
Absorption	ka	h-1	20.3	6.62
Elimination	Ke	L/h	1.02	7
distribution volume	V	L	0.446	4.14
Prop residual error	RUV	%	16.3	
Inter-individual variability in CL	IIV Ke	%CV	43.2	var shrinkage (%) 5.03118691

Table S1: Parameter estimates and variances for the final pharmacokinetic model
RSE: relative standard error, RUV: residual unexplained variability, CV: coefficient of variation.

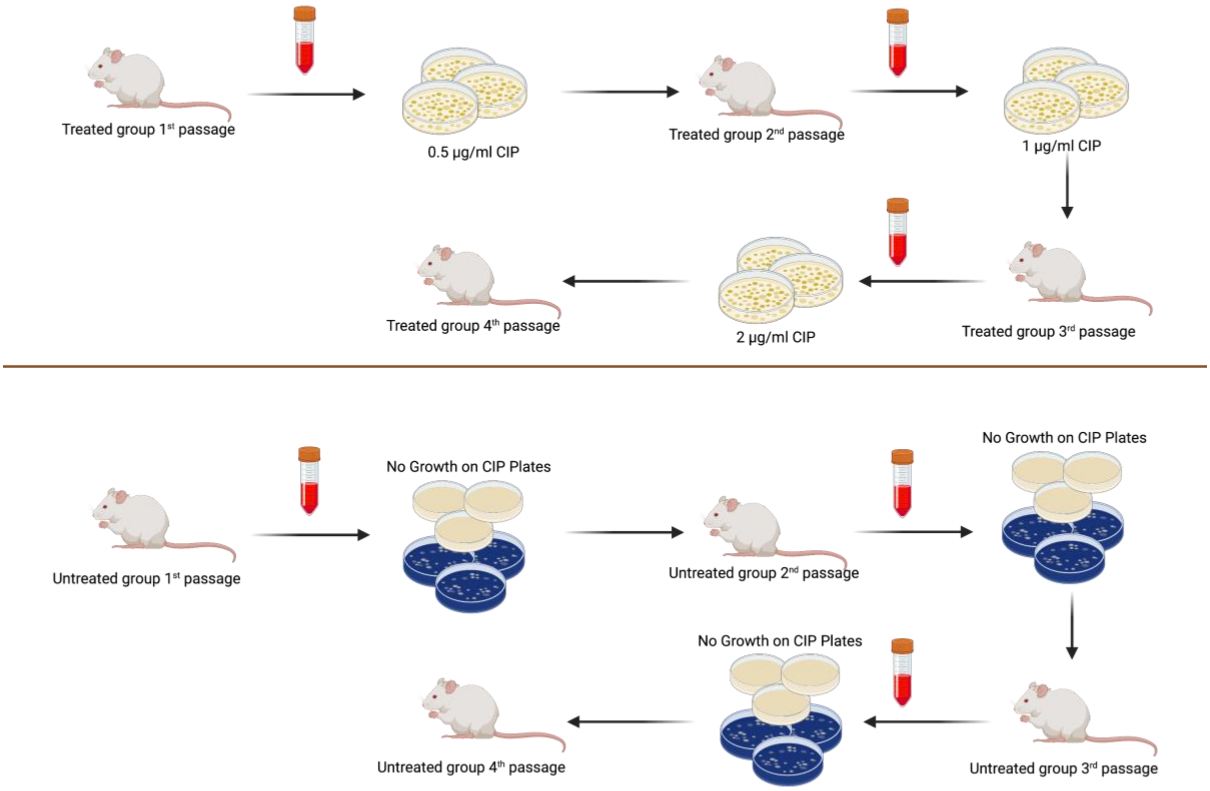

Fig. S1: The figure illustrates the population analysis done for both the treated and control groups. Treated group: after the first passage, the lung homogenate was grown on *P. aeruginosa* isolation agar plates supplemented with CIP at different concentrations (0.5, 1, and 2 µg/ml). The colonies used to inoculate the new passage were collected from the growth on the highest CIP plates which were (0, 0.5, 1, and 2 ml/L, respectively for the 1st, 2nd, $3^{rd,}$ and 4th passage). The control group: we didn't observe any growth on the CIP plates in the different evolutionary passages, so we collected the colonies from the normal counting plates after each passage to start the new inoculum for the control group. The illustration was created with BioRender.com

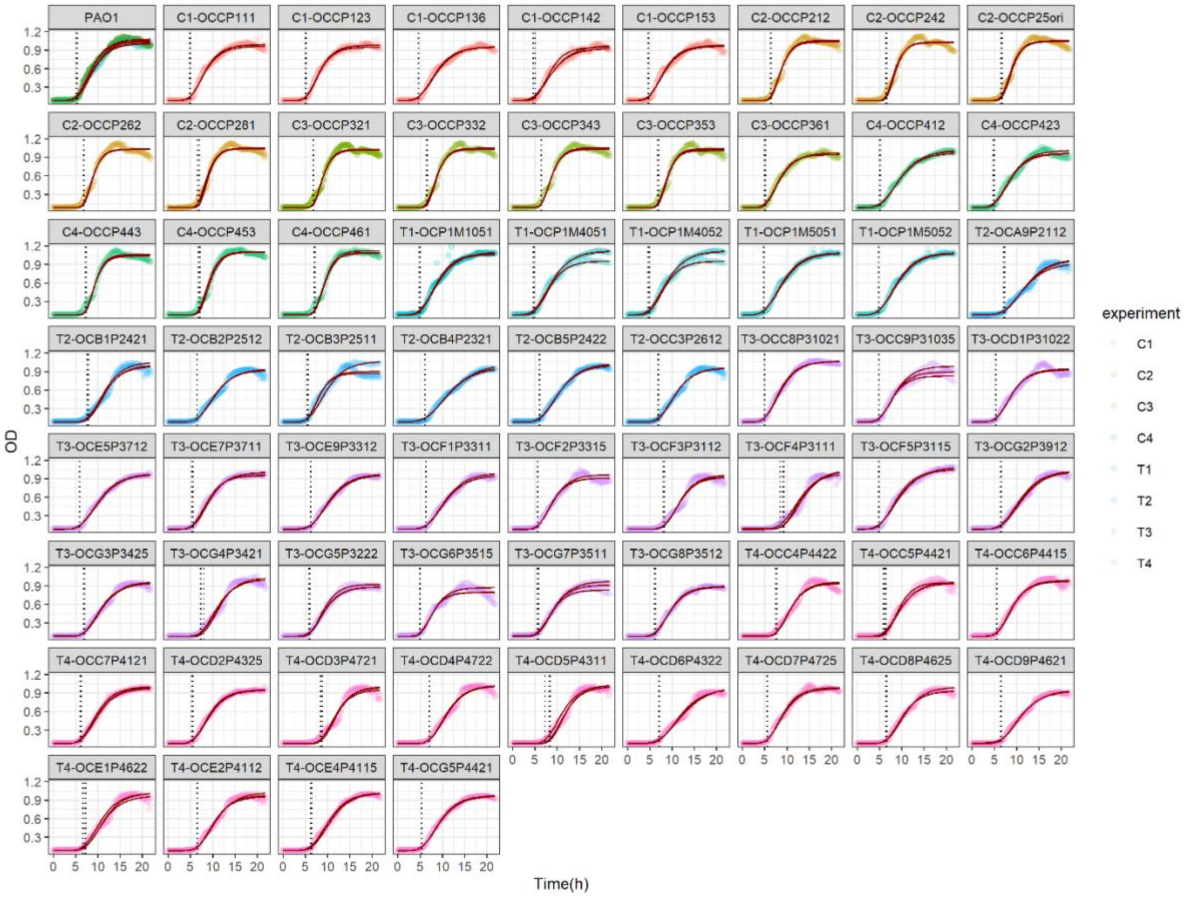

Fig S2: Observed (circles) and fitted (solid line) growth curves, using a Gompertz model for individual strains. The dashed line showed the estimated lag time. Isolates are presented on the strip with the experiment number.

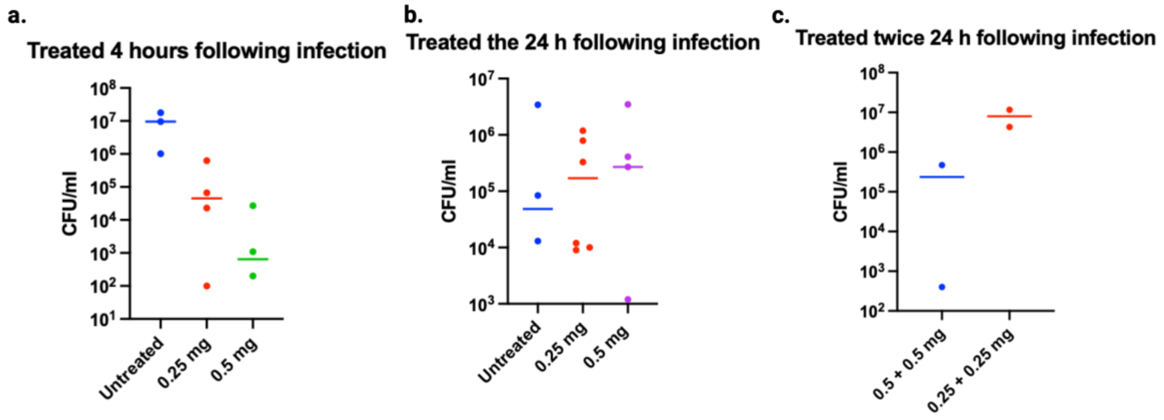

Fig. S3 The figure shows a summary of CFU counts obtained from different pilot studies with different treatments of CIP and different treatment days. a. The mice were treated the same day of infection (4 hours following infection) using 0.25 and 0.5 mg CIP b. The mice were treated 24 hours following infection using 0.25 and 0.5 mg CIP c. the mice were treated 24 hours following infection (twice with 8-hour intervals), either using 0.5 or 0.25 mg CIP.

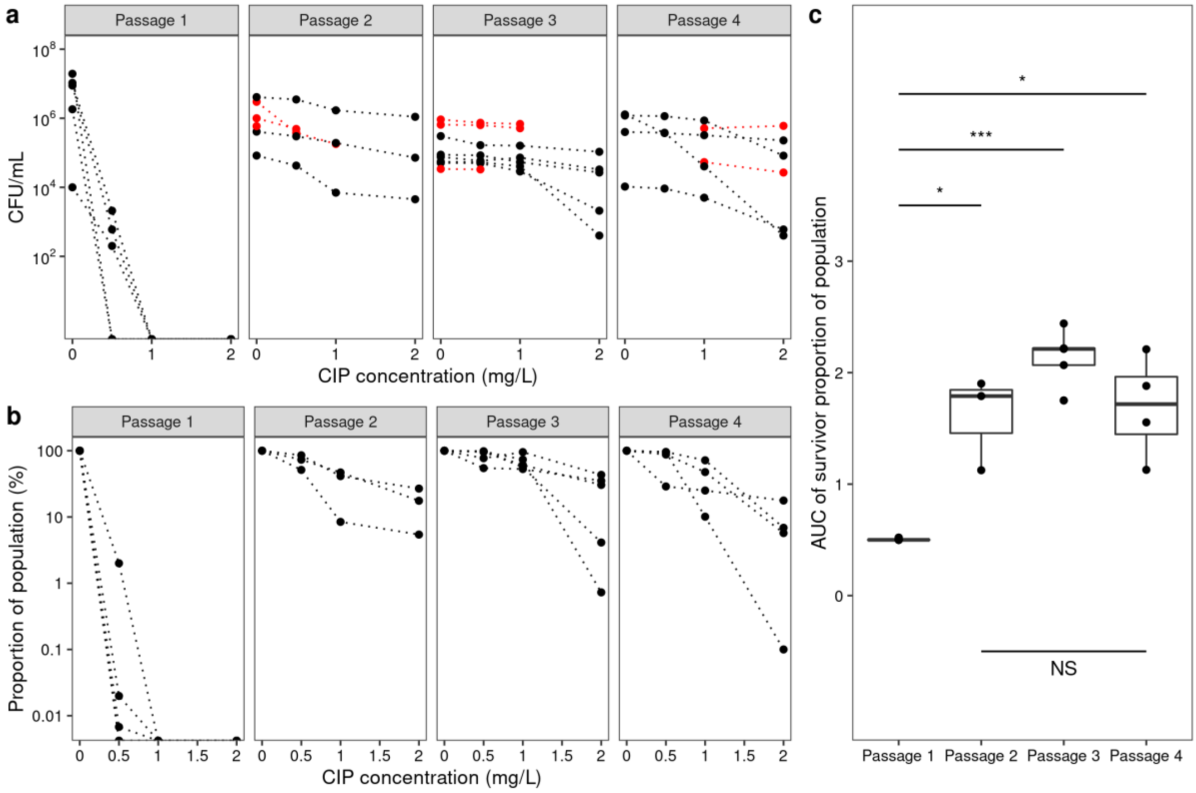

Fig S4: CFU/ml of the quantitative bacteriology is presented as the bacteria from the lung homogenates in each passage were serially diluted and plated on LB agar plates with different concentrations of CIP (0.5,1, and 2 mg/L). **a.** The survival fraction is represented per passage in total CFU/ml and contains full raw CFU data. The red dots marked the uncompleted CIP –-population analysis profile, which was excluded from fraction survival analysis **b.** Represents the percentage of the population survival fraction per passage. **c.** shows the Area-under-the-curve (AUC) under the survival fraction curves from the PAP analysis at (Fig. S4b), quantifying overall resistance per passage. Significant differences were observed between the first passage when compared to other passages (t-test, $p<0.05$). The pairwise t-test p-value of AUC is mentioned in Table S1, the area under the curve is calculated individually for each line from **b**, then we obtained individual AUC for each line in each passage **c**. Each passage's AUCs are then statistically compared – table S1.

	Passage 1	Passage 2	Passage 3	Passage 4
Passage 1	1.0000	0.0452	0.0001	0.0142
Passage 2	0.0452	1.0000	0.1438	0.8043
Passage 3	0.0001	0.1438	1.0000	0.1520
Passage 4	0.0142	0.8043	0.1520	1.0000

	Passage 1	Passage 2	Passage 3	Passage 4
Passage 1	1			
Passage 2	0.0452*	1		
Passage 3	0.0001*	0.1438	1	
Passage 4	0.0142*	0.8043	0.1520	1

*statistically significant difference at p<0.05

Table S2: pairwise t-test p-value of AUC. Each passage's area under the curve (AUC), as depicted in Figure S4c, was systematically paired and statistically compared with every other passage using a t-test. (e.g. passage1 vs passage2 = 0.0452, passage1 vs passage 3 = 0.0001).

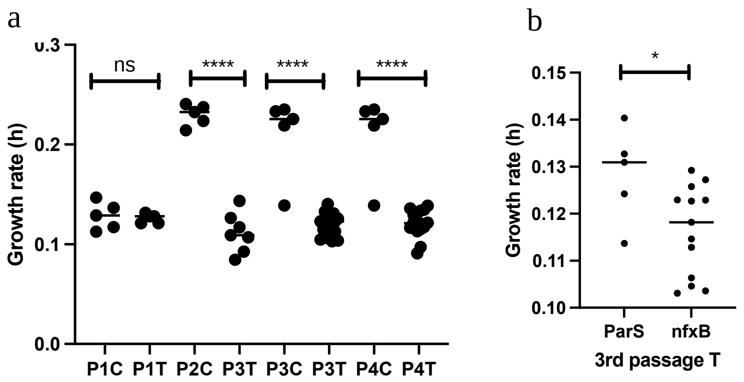

Fig. S5: Growth rate calculated from growth curves for control and CIP-treated isolates. The statistics were done by GraphPad Prism 9 using an unpaired t-test, **** ($p<0.0001$), *($p<0.0324$).

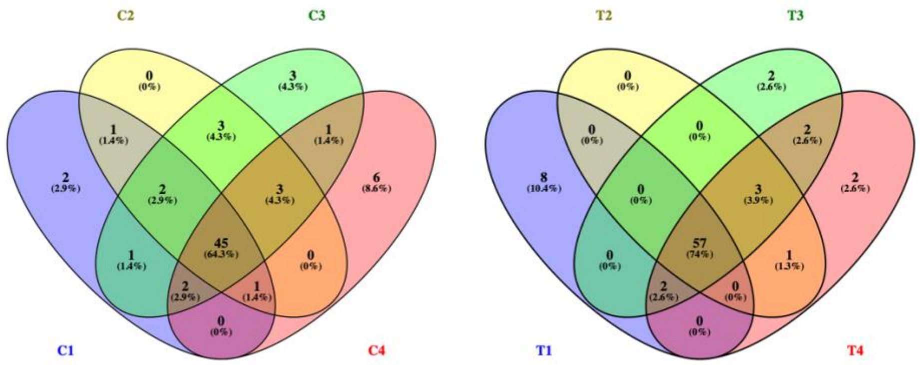

Fig. S6: Variants calling for selected isolates. The Venn diagram depicts the intersection of shared variants throughout the evolution experiment, as well as the distinct variants exclusive to each individual passage in the two groups. The diagram is generated through Venny 2.1[17]. The data represent the variants' positions in the genome.

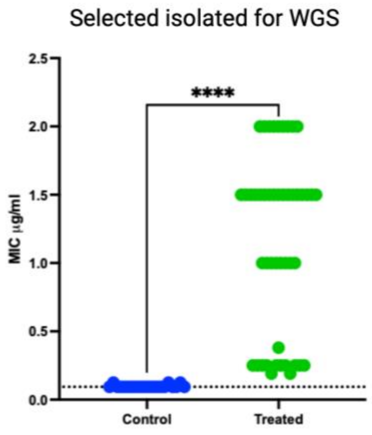

Fig. S7: The CIP MICs for selected isolates used in the whole genome sequencing WGS (control vs CIP-treated) groups. The statistics were done by GraphPad Prism 9 using an unpaired t-test, **** ($p<0.0001$).

Group/ Isolates (No./total in passage)	SNP position	Gene ID	Allele	Annotation	nt. & a.a position change	Function & gene name
**T1 (2/5)**<br>OCP1M1051<br>OCP1M1052	1951314	PA1798	T	Missense_variant<br>(SNP)	c.412G>A<br>p.Ala138Thr	Sensory histidine kinase **ParS**
**T3 (5/18)**<br>OCC8P31021<br>OCC9P31035<br>OCF2P3315<br>OCF5P3115<br>OCG6P3515<br>**T4 (6/16)**<br>OCC4P4422<br>OCC5P4421<br>OCC7P4121<br>OCD7P4725<br>OCD8P4625<br>OCE4P4115	1951435	PA1798	CG	Disruptive_inframe_deletion<br>(Deletion)	c.279_290delCCTCCTCGACAA<br>p.Leu94_Lys97del	Sensory histidine kinase **ParS**
**T1 (2/5)**<br>OCP1M4051<br>OCP1M4052	2213000	PA2020	CG	Frameshift_variant<br>(Deletion)	c.325_337delCTCTACCTCAAGT<br>p.Leu109fs	Transcriptional repressor of **mexXY** operon, **MexZ**
**T1(1/5)**<br>OCP1M5051	2213278	PA2020&PA2021	GT	bidirectional_gene_fusion<br>(Deletion)	n.2213280_2213319delTCTCC<br>CTACCTGTTGCTGGCGGACGCC	Transcriptional repressor of

					TGAGGGCGTCAAT	**mexXY** operon, **MexZ**
**T1 (2/5)**<br>OCP1M1051<br>OCP1M1052	3436431	PA3064	G	Upstream_gene_variant (SNP)	c.-2540T>C	**PelA** An enzyme involved in Pel polysaccharide synthesis.
**T1 (1/5)**<br>OCP1M1052	5125645	PA4577	A	missense_variant (SNP)	c.287C>T<br>p.Ala96Val	RNA polymerase-binding transcription factor DksA
**T2 (7/7)**<br>OCA9P2112<br>OCB1P2421<br>OCB2P2512<br>OCB3P2511<br>OCB4P2321<br>OCB5P2422<br>OCC3P2612<br>**T3 (13/18)**<br>OCD1P31022<br>OCE5P3712<br>OCE7P3711<br>OCE9P3312<br>OCF1P3311<br>OCF3P3112	5155989	PA4600	GC	frameshift_variant (Deletion)	c.430_455delTTCACCGAA CTGTTCATCACCCTGGT<br>p.Phe144fs	Transcriptional regulator NfxB

OCF4P3111						
OCG2P3912						
OCG3P3425						
OCG4P3421						
OCG5P3222						
OCG7P3511						
OCG8P3512						
**T4 (10/16)**						
OCC6P4415=1						
OCD2P4325=1						
OCD3P4721=1						
OCD4P4722=1						
OCD5P4311=1						
OCD6P4322=1						
OCD9P4621=1						
OCE1P4622=1						
OCE2P4112=1						
OCG5P4421=1						
**C2 (5/5)**	5641144	PA5017	TG	frameshift_variant (Deletion)	c.139_148delTGCGAGCGCC p.Cys47fs	Sensory box/GGDEF family protein **DipA**
OCCP212=1						
OCCP242=1						
OCCP25ori=1						
OCCP262=1						
OCCP281=1						
**C3 (4/5)**						
OCCP321						
OCCP321						
OCCP343						
OCCP353						
**C4 (2/5)**						
OCCP443						

OCCP443						
**C3 (1/5)**<br>OCCP321<br>**C4 (2/5)**<br>OCCP443<br>OCCP453	4929573	PA4398	A	missense_variant<br>(SNP)	c.1576C>A<br>p.Leu526Met	Sensory box histidine kinase
**C3 (1/5)**<br>OCCP361	3575749	PA3181	C	upstream_gene_variant<br>(SNP)	c.-4146A>G	Aldose 1-epimerase<br>Carbohydrate-selective porin OprB<br>**edaA**
**C1 (1/5)**<br>OCCP153<br>**T1 (1/5)**<br>OCP1M5051	800889	PA0732	CTGAGGGTGAGGGT	conservative_inframe_insertion	c.243_244insACCCTC<br>p.Ser81_Thr82insThrLeu	L,D-transpeptidase
**T4 (1/16)**<br>OCG5P4421	1844903	PA1695	TGTTGGCGTTGGCGT<br>TGGCGTTGGCGTTGGC<br>GTTGGCGTTGGCGTTG<br>GCGTTGGCGTTGG<br>CGTTGGCGTTGGCG	conservative_inframe_insertion<br>(Insertion)		pscP<br>Type III secretion protein (YscP)

**T4 (1/16)** OCG5P4421	2186926	PA1995	CGCGCGGCCGGCGCCT	upstream_gene_variant (Insertion)	YjbR-like superfamily

Table S3: Detailed annotation information of the discussed variants in the study. Group names: T1= Passage 1 treated with CIP, T2= Passage 2 treated with CIP, T3= Passage 3 treated with CIP, T4= Passage 4 treated with CIP. C1= Passage 1 Control (Treated with Saline), C2 = Passage 2 Control (Treated with Saline), C3= Passage 3 Control (Treated with Saline), C4= Passage 4 Control (Treated with Saline). The number of isolates where the mutation exists is specified (no. of mutated isolates/total number of sequenced isolates within the group).
